# Cell-Permeable Succinate Rescues Mitochondrial Respiration in Cellular Models of Statin Toxicity

**DOI:** 10.3390/ijms22010424

**Published:** 2021-01-03

**Authors:** Vlad F. Avram, Imen Chamkha, Eleonor Åsander-Frostner, Johannes K. Ehinger, Romulus Z. Timar, Magnus J. Hansson, Danina M. Muntean, Eskil Elmér

**Affiliations:** 1 Department of Internal Medicine-Diabetes, Nutrition and Metabolic Diseases, “Victor Babeș” University of Medicine and Pharmacy Timișoara, Romania, Eftimie Murgu Sq. No. 2, 300041 Timișoara, Romania; avram.vlad@umft.ro (V.F.A.); timarrz@yahoo.com (R.Z.T.); 2 Center for Translational Research and Systems Medicine, “Victor Babeș” University of Medicine and Pharmacy Timișoara, Romania, Spl. Tudor Vladimirescu No. 14, 300173 Timișoara, Romania; 3 Mitochondrial Medicine, Department of Clinical Sciences Lund, Faculty of Medicine, Lund University, BMC A13, 221 84 Lund, Sweden; imen.chamkha@med.lu.se (I.C.); eleonor.asander_frostner@med.lu.se (E.Å.-F.); johannes.ehinger@med.lu.se (J.K.E.); magnus.hansson@med.lu.se (M.J.H.); 4 Abliva AB, Medicon Village, 223 81 Lund, Sweden; 5 Department of Functional Sciences-Pathophysiology, 2Center for Translational Research and Systems Medi-cine, “Victor Babeș” University of Medicine and Pharmacy Timișoara, Romania, Eftimie Murgu Sq. No. 2, 300041 Timișoara, Romania

**Keywords:** platelets, HepG2 cells, statins, cell-permeable succinate, NV118, mitochondria

## Abstract

Statins are the cornerstone of lipid-lowering therapy. Although generally well tolerated, statin-associated muscle symptoms (SAMS) represent the main reason for treatment discontinuation. Mitochondrial dysfunction of complex I has been implicated in the pathophysiology of SAMS. The present study proposed to assess the concentration-dependent ex vivo effects of three statins on mitochondrial respiration in viable human platelets and to investigate whether a cell-permeable prodrug of succinate (complex II substrate) can compensate for statin-induced mitochondrial dysfunction. Mitochondrial respiration was assessed by high-resolution respirometry in human platelets, acutely exposed to statins in the presence/absence of the prodrug NV118. Statins concentration-dependently inhibited mitochondrial respiration in both intact and permeabilized cells. Further, statins caused an increase in non-ATP generating oxygen consumption (uncoupling), severely limiting the OXPHOS coupling efficiency, a measure of the ATP generating capacity. Cerivastatin (commercially withdrawn due to muscle toxicity) displayed a similar inhibitory capacity compared with the widely prescribed and tolerable atorvastatin, but did not elicit direct complex I inhibition. NV118 increased succinate-supported mitochondrial oxygen consumption in atorvastatin/cerivastatin-exposed platelets leading to normalization of coupled (ATP generating) respiration. The results acquired in isolated human platelets were validated in a limited set of experiments using atorvastatin in HepG2 cells, reinforcing the generalizability of the findings.

## 1. Introduction

Atherosclerosis is the major pathomechanism of ischemic cardiovascular diseases that together with stroke are the leading causes of global mortality [1]. According to Mach et al. [2], the current guidelines for the management of dyslipidemias recommend more stringent targets for lipid control in these pathologies; in this respect, statins are the first-line treatment for regulating cholesterol levels in cardiac and metabolic diseases. Statins reduce the liver synthesis and increase plasma clearance of LDL-cholesterol by inhibiting hydroxymethylglutaryl-coenzyme A (HMG-CoA) reductase, the rate-controlling enzyme in cholesterol biosynthesis [3]. Despite the fact that statins are generally well tolerated (with plasma concentrations normally in the nanomolar range [4]), a limited number of side-effects have been reported in the literature. Among these, statin-associated muscle symptoms (SAMS) that vary in intensity from weakness and myalgias to myopathy and in most severe (yet very rare) cases, rhabdomyolysis, are most frequently encountered [5,6].

Mitochondrial dysfunction has been systematically reported to play an important role in the pathophysiology of SAMS [7,8,9]. Thus, statins interfere with mitochondrial function in a concentration-dependent manner in various cell lines, animal and human tissue samples either due to the HMG-CoA inhibition or off-target effects. With respect to the latter, varied and complex interference with the activity of the electron transport system (ETS) and mitochondrial content have been reported in skeletal muscle [9,10,11,12,13].

In the past decade, peripheral blood platelets have been increasingly used as a source of viable mitochondria to investigate cellular respiratory dysfunction in order to complement studies reporting organ-related mitochondrial dysfunction in various pathologies [14]. Due to the low invasiveness through which they are obtained (especially as compared to muscle cells), platelets are an attractive source of human mitochondria for studies of toxic effects elicited by statin exposure [15,16].

In the literature, a frequently reported statin-related impairment of the ETS is the inhibition of NADH-linked respiration [8,17]. In order to bypass this complex I defect, enhancing complex II (succinate)-dependent respiration could be a viable approach. However, the dicarboxylic acid succinate displays a very limited cellular uptake, a disadvantage that has been recently overcome by using cell-permeable succinate prodrugs in various conditions associated with impairment of NADH-supported respiration [18,19,20].

The present study performed in isolated human platelets was aimed to: (i) evaluate and localize the ETS inhibition of three statins: atorvastatin, simvastatin (as the mostly prescribed lipophilic statins) and cerivastatin (as a positive control of toxicity, since it was withdrawn from the market in 2001 due to fatal cases of rhabdomyolysis [21]), (ii) assess the drug effects on adenosine triphosphate (ATP)-generating (oligomycin-sensitive) mitochondrial respiration and (iii) investigate whether NV118, a cell-permeable succinate prodrug, can compensate for the statin-induced mitochondrial dysfunction. Since the pleiotropic effects of statins were reported to occur at micromolar concentrations, we chose to titrate them in such concentrations in order to assess the potency of the novel prodrug.

## 2. Results

### 2.1. Statins Decreased Mitochondrial Respiration in Intact Platelets in a Concentration-Dependent Manner

**Figure 1:**
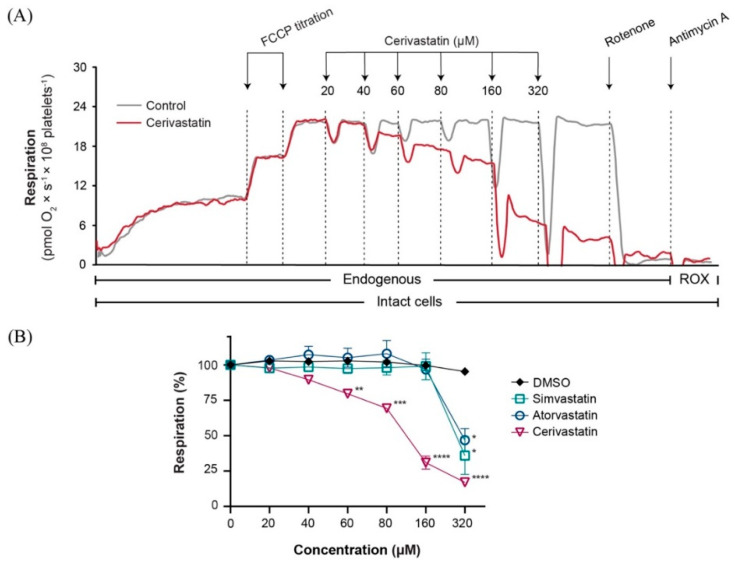
Statins induced a concentration−dependent inhibition of mitochondrial respiration in intact human platelets. (**A**) Representative traces of cerivastatin (red) and DMSO (grey) titration. Respiration of human platelets was measured after sub-maximal uncoupling with FCCP (2 µM). (**B**) The concentration-dependent effect of simvastatin (open square), atorvastatin (open circle) and cerivastatin (open triangle) was measured by titrating increasing concentrations of statin or vehicle (DMSO) (black rhombus). Data is expressed as mean±SEM. FCCP, carbonyl cyanide p-(trifluoromethoxy) phenylhydrazone; DMSO, dimethyl sulfoxide; ROX, residual oxygen consumption. Two-way ANOVA with Bonferroni post hoc test was performed on antimycin-corrected data. * *p* < 0.05; ** *p* < 0.01; *** *p* < 0.001; **** *p* < 0.0001 vs. DMSO.

The effect on mitochondrial respiration with increasing concentrations up to 320 µM of three different statins was evaluated in intact human platelets. Significant respiratory inhibition was elicited by all three statins when applied in the highest concentration, reducing mitochondrial respiration to 35.9% ± 13, 46.8% ± 8 and 17.0% ± 2 of baseline for simvastatin, atorvastatin and cerivastatin, respectively (Figure 1B). Cerivastatin significantly decreased mitochondrial respiration in intact platelets starting from the concentration of 60 µM (79.6% ± 2).

### 2.2. Statins Induced Mitochondrial Dysfunction in Permeabilized Platelets via Several Distinct Mechanisms

In order to further dissect the mechanisms of statin-related ETS impairment, the concentration-dependent effect of three concentrations (40, 80 and 160 µM) of each statin on mitochondrial respiration was evaluated in permeabilized platelets.

**Figure 2:**
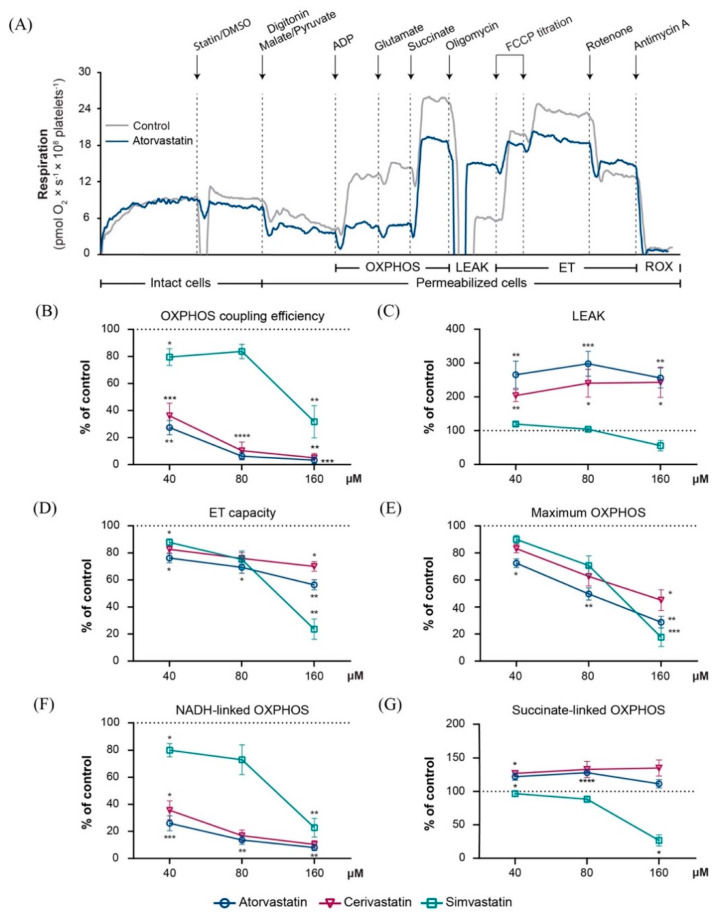
Statins induced a concentration−dependent inhibition of mitochondrial respiration in permeabilized human platelets. (**A**) Representative traces of atorvastatin 40 µM (blue) and DMSO (grey). Concentration-dependent effects were assessed for 3 concentrations (40 µM, 80 µM and 160 µM, respectively) of atorvastatin (open circle), cerivastatin (open triangle) and simvastatin (open square), respectively. OXPHOS coupling efficiency (**B**), LEAK (**C**), maximal ET capacity (**D**), maximal OXPHOS (**E**), NADH-linked OXPHOS (**F**), and succinate-linked OXPHOS (**G**) capacities were evaluated. Data is expressed as mean±SEM of the percent of control (platelets exposed to the corresponding volume of DMSO for each of the 3 concentrations of statin). Two-way ANOVA with Bonferroni post hoc test was performed on antimycin-corrected data. DMSO, dimethyl sulfoxide; ET, electron transport; LEAK, non-phosphorylating resting stat; OXPHOS, oxidative phosphorylation; ROX, residual oxygen consumption. * *p* < 0.05; ** *p* < 0.01; *** *p* < 0.001; **** *p* < 0.0001 vs. DMSO.

Oxidative phosphorylation (OXPHOS) coupling efficiency is a measure of the ATP generating efficiency, and was calculated according to a preestablished formula (1-LEAK/maximal phosphorylating respiration) [22]. The OXPHOS coupling efficiency integrates both uncoupling (increased LEAK) and electron transport (ET) inhibition which work additively to limit ATP production. The coupling efficiency was significantly decreased already at the lowest concentration tested (40 µM) to 36% ± 9 (*p* < 0.001), 27% ± 5 (*p* < 0.01) and 80% ± 6 (*p* < 0.05) of control for cerivastatin, atorvastatin, and simvastatin, respectively (Figure 2B) reaching: 5% ± 3 (*p* < 0.01), 3.3% ± 2.4 (*p* < 0.001), and 31.7% ± 11.9 (*p* < 0.01) at 160 µM.

LEAK respiration (increase in the non-ATP generating oxygen consumption, i.e., uncoupling) was significantly increased for cerivastatin and atorvastatin but not for simvastatin (Figure 2C). All three statins presented a concentration-dependent reduction of the ET capacity and maximum OXPHOS that varied with each statin. At the highest concentration (160 µM), platelet mitochondria ET capacity was significantly reduced to 70% ± 4 by cerivastatin (*p* < 0.05), 56.3% ± 4 by atorvastatin (*p* < 0.01) and 23.6% ± 7 by simvastatin (*p* < 0.01) compared to control (Figure 2D) and maximum OXPHOS was reduced to 45% ± 8 by cerivastatin (*p* < 0.05), 28.8% ± 4 by atorvastatin (*p* < 0.01) and 17.6% ± 7 by simvastatin (*p* < 0.001) as compared to control (Figure 2E).

When examining the NADH (complex I) and succinate (complex II) pathways separately (Figure 2F,G), when applied in the lowest concentration (40 µM), simvastatin elicited the mildest reduction in NADH-dependent OXPHOS (80% ± 5 of control) of the three statins. At the same concentration, both atorvastatin and cerivastatin significantly inhibited NADH-linked respiration (25.9% ± 6 and 35.5% ± 7 of control, respectively), and the inhibition was further augmented (respiration decreased) for the higher concentrations. Also, when applied in the highest concentration (160 µM) simvastatin (but not atorvastatin or cerivastatin) significantly decreased the succinate-dependent OXPHOS (Figure 2G) by 26.7% ± 8. To be noted, atorvastatin and cerivastatin did not inhibit succinate-supported respiration. The elevated succinate-linked OXPHOS respiration values for atorvastatin and cerivastatin at 80 µM (Figure 2G) are likely related to increased uncoupling (increased LEAK), as demonstrated for these statins (Figure 2C).

### 2.3. Simvastatin and Atorvastatin (but Not Cerivastatin) Directly Inhibited Complex I-Linked Respiration

**Figure 3:**
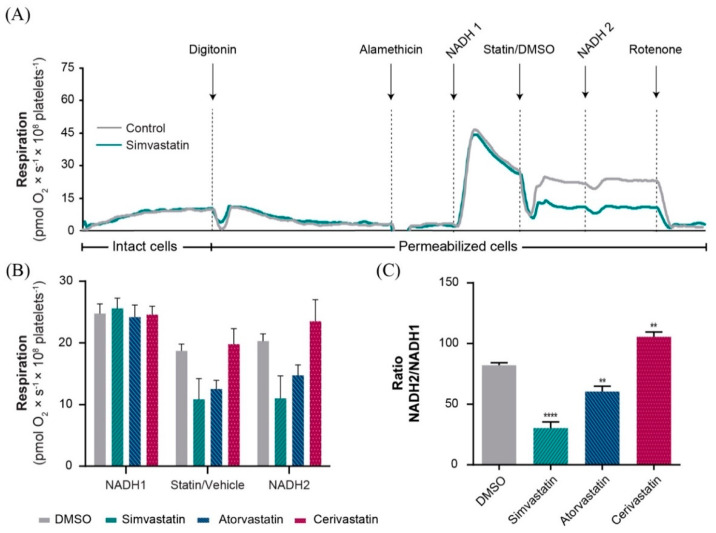
Statin effects on NADH−linked oxygen consumption in alamethicin-permeabilized platelet mitochondria. (**A**) Representative trace of simvastatin 160 µM (green) and DMSO (grey). (**B**) Oxygen consumption of permeabilized platelet mitochondria was measured at three points: Figure 0. mM) next in the presence of the statin (160 µM)/DMSO control (DMSO-grey bar, simvastatin-green bar, atorvastatin-blue bar, cerivastatin-red bar) and then after the addition of statin plus a second dose of NADH (0.75 mM). (**C**) Calculated NADH-linked oxygen consumption (∆) for each statin and DMSO control, evaluated as the ratio between the two consecutive additions of NADH (NADH2/NADH1). Data is expressed as mean ± SEM of rotenone-corrected data, one-way ANOVA with Bonferroni post hoc test. DMSO, dimethyl sulfoxide. ** *p* < 0.01; **** *p* < 0.001 vs. DMSO.

We further specifically evaluated the effect of statins on NADH-linked mitochondrial oxygen consumption to investigate whether the decrease in NADH-linked respiration observed (Figure 2E) was due to a direct inhibition of complex I (NADH-dehydrogenase) or related to an upstream effect limiting the supply of NADH to complex I. To provide access of NADH to complex I of mitochondria, platelets and their mitochondria were permeabilized with digitonin and alamethicin (a 20-amino acid channel-forming peptide antibiotic), respectively. The respiratory activity of complex I was calculated as a ratio of respiration of two additions of NADH (before and after 160 µM statin/DMSO addition) (Figure 3A). Both simvastatin and atorvastatin elicited a significant decrease in NADH-linked oxygen consumption (*p* < 0.001 and *p* < 0.01, respectively), with a more severe reduction for simvastatin (Figure 3C). In contrast, cerivastatin did not inhibit NADH-induced oxygen consumption in the permeabilized mitochondria. In fact, in the presence of cerivastatin, NADH-induced oxygen consumption was significantly increased. This observation suggests that cerivastatin elicits mitochondrial toxicity via a mechanism different from direct complex I inhibition.

### 2.4. Cell-Permeable Succinate Bypassed the NADH-Linked Mitochondrial Dysfunction Induced by Statins in Human Platelets

**Figure 4:**
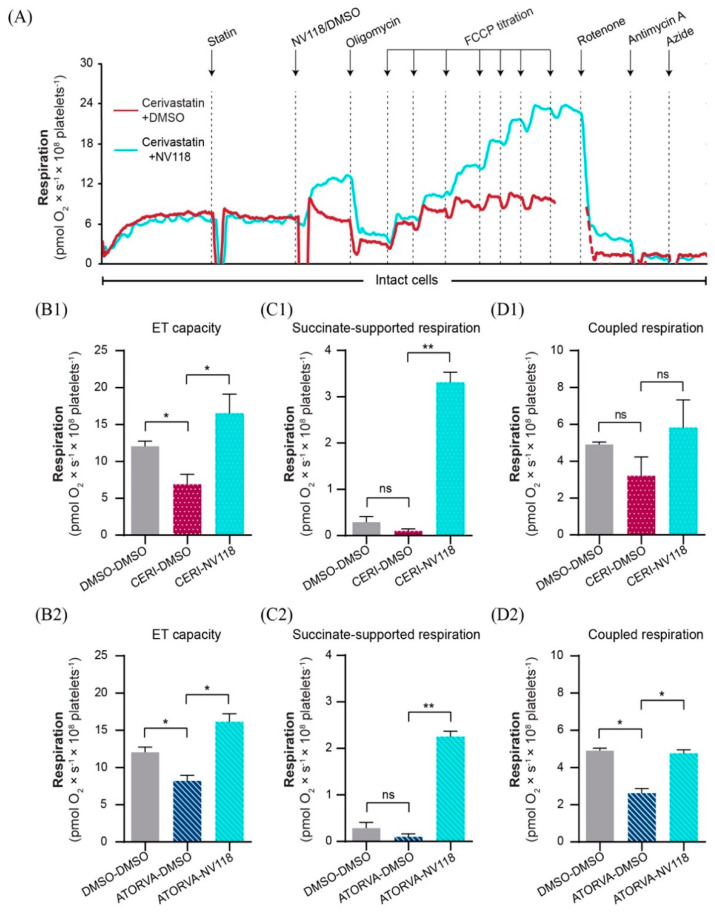
The effects of NV118 on statin−dependent respiratory dysfunction in human platelets. (**A**) Representative overlay trace of statin-exposed platelets in the absence (red) or presence (blue) of the succinate prodrug NV118. NV118 effects in cerivastatin (**B1**,**C1**,**D1**) and atorvastatin (**B2**,**C2**,**D2**) exposed platelets were measured as compared to its vehicle (DMSO). As negative control of the experiment, platelets were exposed only to DMSO (DMSO-DMSO). Data is expressed as mean±SEM. One-way ANOVA with Bonferroni post hoc test was performed on antimycin-corrected data. ATORVA, atorvastatin; CERI, cerivastatin; DMSO, dimethyl sulfoxide; ET, electron transport. ns = no significance; * *p* < 0.05; ** *p* < 0.01.

Since we had demonstrated that cerivastatin and atorvastatin caused mitochondrial dysfunction via the inhibition of NADH-linked respiration (Figure 2A) we further investigated whether the respiratory impairment caused by a concentration of 80 µM of these statins can be alleviated in the presence of the cell-permeable succinate prodrug NV118. A representative overlay trace of statin-exposed platelets in the presence or absence of NV118 is depicted in Figure 4A. The addition of the prodrug to either atorvastatin or cerivastatin-exposed platelets resulted in an increase in the ET capacity of the treated platelets (Figure 4B1,B2) by increasing succinate-supported mitochondrial oxygen consumption (Figure 4C1,C2) leading to levels of coupled respiration similar to those of the control samples (Figure 4D1,D2).

### 2.5. Cell-Permeable Succinate Bypassed Mitochondrial Dysfunction Induced by Statins in HepG2 Cells

**Figure 5:**
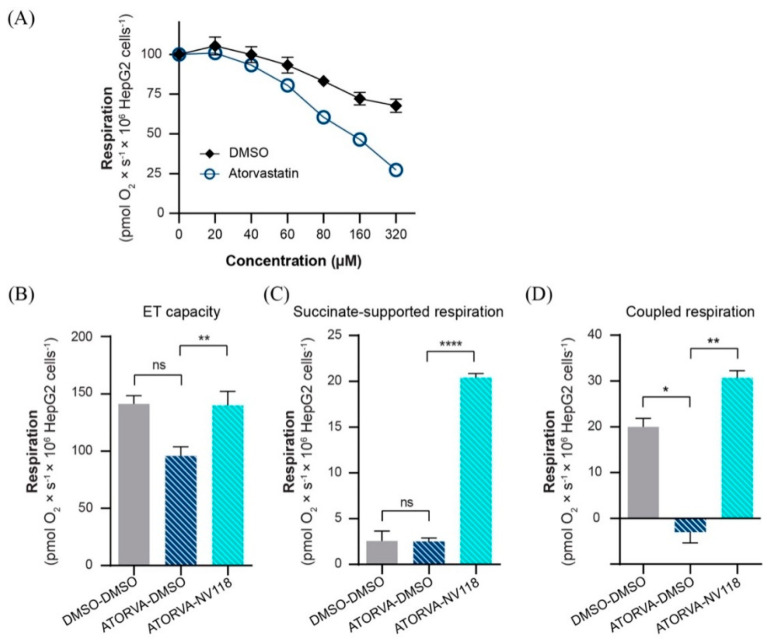
The effects of NV118 on statin−dependent respiratory dysfunction in HepG2 cells. (**A**) Respiration was assessed after sub-maximal uncoupling with FCCP (4 µM) followed by increasing concentrations of atorvastatin or vehicle (DMSO). NV118 effects on atorvastatin exposed HepG2 cells (**B**–**D**) were measured as compared to its vehicle (DMSO). As negative control of the experiment HepG2 cells were exposed only to DMSO (DMSO-DMSO). Data is expressed as mean ± SEM. One-way ANOVA with Bonferroni post hoc test was performed on antimycin-corrected data. ATORVA, atorvastatin; DMSO, dimethyl sulfoxide; ET, electron transport. ns = no significance; * *p* < 0.05; ** *p* < 0.01; **** *p* < 0.0001.

In order to assess whether the effects of succinate can be reproduced in different cells, we selected the human-derived liver cancer cell line HepG2. Oxygen consumption of HepG2 cells was assessed after exposure to the same concentrations of atorvastatin used in the previously described protocol in intact platelets, causing a reduction up to 27% ± 1 of control at 320 µM (Figure 5A). The addition of NV118 to the atorvastatin exposed HepG2 cells elicited results comparable to those obtained in human platelets. In the presence of NV118, ET capacity increased (Figure 5B) by increasing succinate-supported mitochondrial oxygen consumption (Figure 5C). Further, coupled (ATP producing) respiration (calculated as the respiratory rate before and after oligomycin addition) was normalized in NV118-treated samples (Figure 5D).

## 3. Discussion

The main finding of this study is that statins caused a significant reduction of OXPHOS coupling efficiency, i.e., the respiratory capacity to produce ATP, even in the low µM range. The effect was mediated via a two level-impairment of mitochondrial respiration: increased uncoupling (increased LEAK state) and inhibition of electron transport, mostly through the reduction of NADH-linked respiration. The properties of each statin are unique and are summarized in Table 1.

In our study we used human platelets as a source of primary human mitochondria. As platelets are known to rely on OXPHOS they can act as a mirror of mitochondrial function in other tissues. The comparable mitochondrial effects of drug-induced toxicity between human platelets and HepG2 cells we observed, have been previously shown by Piel et al. [18] confirming the suitability of platelets for the study of drug-induced mitochondrial effects.

Our observations are in line with the pioneering study of Kaufmann et al. [23], which reported that exposure of rat myoblasts to cerivastatin, atorvastatin and simvastatin for 24 h at 100 µM resulted in cytotoxicity, inhibition of complexes I, III, IV and decreased mitochondrial membrane potential. However, the uncoupling effects of statins appears to be cell type-dependent. In contrast to our data in freshly isolated human platelets, Kaufman et al. [23] reported that cerivastatin, but not atorvastatin or simvastatin elicited uncoupling in rat myoblasts. More recently, Broniarek et al. [24] reported in mitochondria isolated from a stable human endothelial cell line (EA.hy926 derived from human umbilical vein) an uncoupling effect at concentration up to 100 µM and inhibition of respiration for higher concentrations (up to 300 µM) for atorvastatin (but not for pravastatin). The same group further reported in the same in vitro experimental model that even lower concentrations of atorvastatin (100 nM) elicited a decrease in both maximal respiration (as result of supercomplexes rearrangement) and the total cellular coenzyme Q10 content [24]. Interestingly, these authors also reported a significant increase in the expression of UCP2 protein in mitochondria isolated from the endothelial cells exposed to 100 nM atorvastatin [25]. Vevera et al. [11] showed in an in vivo study that 4-week treatment with simvastatin reduced platelet respiration in rats. Furthermore, the same group also showed in humans that a six-week treatment with statins in therapeutic concentrations resulted in decreased NADH-linked respiration in permeabilized, but not intact human platelets, suggesting some kind of in vivo compensation for the statin effect. The observation that the in vivo effects of statins on NADH-linked respiration might be compensated has been recently proposed by Gvozdjakova et al. [26]. These authors showed that NADH-linked OXPHOS was, in fact, increased in patients with different pathologies, e.g., diabetes, nephropathy, or dialysis, that were treated with statins [26]. Moreover, pharmacokinetics studies have demonstrated that the plasma levels of statins under normal conditions are in the nanomolar range, which strongly suggests that statin therapy does not induce mitochondrial inhibition under normal therapeutic conditions [4,27,28]. In a recent elegant study carried out by the group of Rasmussen [13], the authors reported that chronic treatment with simvastatin resulted in higher respiratory efficiency in both peripheral blood mononuclear cells and platelets and an increase in complex I activity in these blood cells while having as side effect an increased generation of mitochondrial superoxide. While providing evidence that in clinical setting statins are not deleterious for mitochondrial respiration of blood cells, the authors do not exclude the possibility that even in therapeutic doses, statins could decrease the function of mitochondria in skeletal muscle despite improving the one of circulating cells.

In practice, however, situations may occur in which plasma statin concentration is increased beyond the therapeutic range. One study suggests that 60% of cases of statin-induced rhabdomyolysis were associated with drug-drug interactions [29]. Statins are metabolized through cytochrome P450 and when administered with other inhibitors of this pathway, their plasma levels increase [30]. Among the inhibitors of the cytochrome P450 pathway, the most frequently prescribed are: amiodarone, gemfibrozil, calcium channel blockers, cyclosporine, azole antifungals, macrolide antibiotics, and HIV protease inhibitors. Consumption of grapefruit juice in high amounts has also been reported to increase statin concentrations in plasma. All of these are possible concomitant treatment in patients chronically taking statins [2,31,32,33,34]. Diltiazem increased statin plasma levels by a factor of 4 while grapefruit juice in some cases increases it by a factor of 15 [35,36]. Furthermore, it should be noted that statin tissue concentrations differ from their serum concentrations, with muscle levels down to only a third of those in the serum while the liver presents concentrations that are twice as high as in serum [4].

Furthermore, some of the above-mentioned drugs have been reported to cause mitochondrial dysfunction via the inhibition of the NADH-liked respiration by themselves; thus, a cumulative effect is likely to occur when administered together with statins [37,38,39,40]. In this respect, Wagner et al. [41] showed that propranolol had similar effects to statins on mitochondrial respiration and, when the drugs were combined, their effects were additive.

Certain situations have been reported in the literature to increase the risk of developing muscle symptoms in patients using statins. For example, hypothyroidism, a condition where statin therapy is indicated, has been associated with increased risk of myopathy that can be due to a combination of increased oxidative stress and mitochondrial dysfunction reported to occur in this disease [5,42]. Alcohol intake of more than 30 g per day in men or 20 g per day in women was also associated with higher risk of SAMS [43]; in this case, the increase in reactive oxygen species generation by chronic alcohol consumption was associated with a decrease in ATP synthase and NADH-dehydrogenase enzymatic levels [44]. As demonstrated in the present study, statin use alone decreases NADH-linked respiration in a concentration-dependent manner. Also, there is a difference between the concentrations which cause mitochondrial impairment in intact and permeabilized cells. Since the three studied statins are all lipophilic [7] it is more likely that the acute experiments carried out in the permeabilized setting are closer to the situation of chronic treatment, where the statins have ample time to permeate the cells. The toxic effects on skeletal muscle have been mostly associated with high doses of lipophilic statins (e.g., atorvastatin and simvastatin) and less with the hydrophilic ones (e.g., pravastatin and rosuvastatin) [45].

Age must also be taken into account as SAMS tend to be more frequent in elderly people (over 80) [5]. Since advanced age determines enzymatic changes that lead to lower oxidative capacity and ATP generation (per mitochondrial volume) [46], decreased OXPHOS coupling efficiency (actual ATP production) by statins, and possibly other concomitant treatment, could elicit SAMS in these patients.

Most of the literature data on statin-induced mitochondrial dysfunction report a direct effect on complex I with the reduction of NADH-linked respiration [10,45,47]. The severe impairment of NADH-linked respiration elicited by titration of cerivastatin in intact cells demonstrated here was possibly not a surprise since this statin was early withdrawn from the market due to several lethal cases of rhabdomyolysis [21]. However, in permeabilized cells the inhibition of NADH-linked respiration and coupling efficiency (ATP production) was comparable to that of atorvastatin. One of the most intriguing findings unraveled by the present study is that the reduction of NADH-linked respiration induced by cerivastatin originates upstream of complex I itself, limiting the supply of NADH to complex I. This could be an explanation as to why cerivastatin caused such an alarming number of lethal cases of rhabdomyolysis, which led to its withdrawal from the market [21] while simvastatin and atorvastatin are still in current use. However, the underlying mechanism remains to be determined.

Judging by the high concentrations required to induce mitochondrial dysfunction with statins, most probably mitochondrial impairment is only one of the several mechanisms through which statin-induced myopathy occurs [45]. To name a few possibilities: inhibition of carnitine palmitoyltransferase I (CPT-I) which leads to decreased mitochondrial β-oxidation (fatal condition) or inhibition of the mevalonate pathway causing decreased availability of geranylgeranyl-pyrophosphate and impairment of ATP production via decreasing mitochondrial membrane potential [23,48,49,50].

Statin use has also been linked with reduced plasma levels of vitamin D [51], since the main source of vitamin D is provided by the conversion of 7-dehydrocholesterol (which requires cholesterol for biosynthesis) under the influence of UV radiation [52]. Low levels of vitamin D, apart from osteomalacia, also cause muscle weakness and myopathy [52]. Supplementation of vitamin D has been reported to improve statin tolerance [53]. Vitamin D supplementation could very well be a good way to improve statin adherence, by reducing SAMS, however this data has yet to be confirmed by large scale randomized controlled trials [5,52].

Ubiquinone (coenzyme Q_10_) deficiency is the direct consequence of isoprenois intermediates depletion in the mevalonate pathway associated with HMG-CoA inhibition by statins and has been initially considered the most relevant pathomechanism for the induction of SAMS [8,10]. Whereas early isolated case reports indicated a decrease in muscle CoQ in statin-treated patients, data from the recent LIFESTAT study showed that both muscle content and plasma level of CoQ10 were comparable in simvastatin-treated patients with and without myalgia [54]. However, previous studies reported that plasma levels of ubiquinone are decreased in statin-treated patients by approximately 0.5 μmol/L, regardless the type of statin used. This level of ubiquinone represents somewhere between 30–40% of the total ubiquinone serum levels under normal conditions. One explanation is that since a considerable amount of ubiquinone is bound to LDL-cholesterol, levels of ubiquinone go down as LDL-cholesterol is diminished by the statin [9]. At variance, in the cohort of simvastatin-treated patients reported by Durhuus et al. [13], a 5-fold increase of serum ubiquinone has been reported. However, supplementation of CoQ10 in patients treated with statins offered conflicting results regarding the benefit in reduction of SAMS or CK levels [9] or superoxide production by blood cells (regardless the presence of SAMS) [13]. This is most probably due to the fact that serum and muscle ubiquinone levels are differently modulated and, since the serum levels of ubiquinone did not correlate with the activities of the ETS enzymes, CoQ should not be used as a marker when assessing the effects of statins on energy metabolism [55]. Instead, the level of mitochondrial superoxide in peripheral blood cells has recently emerged as putative biomarker for SAMS [13].

**Figure 6:**
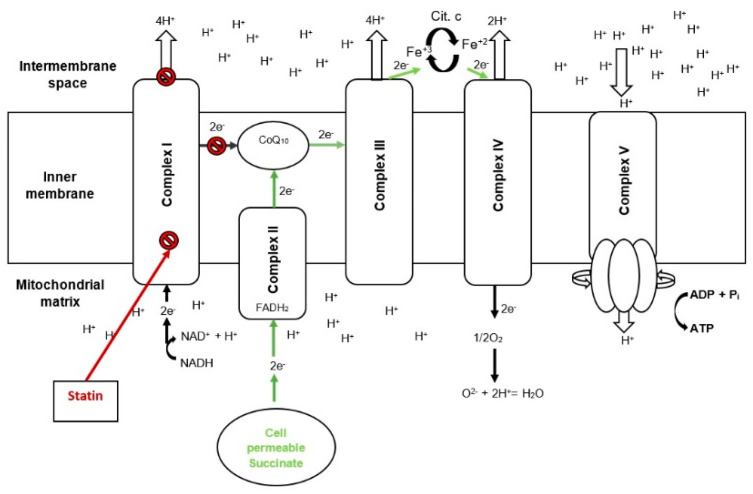
Cell permeable succinate bypasses statin−induced complex I mitochondrial dysfunction. Scheme 10. ubiquinone; e^−^, electron; FADH_2_, dihydroflavine-adenine dinucleotide; Fe^+3^, iron(III); Fe^+2^, iron(II); H^+^, proton; NAD^+^, Nicotinamide adenine dinucleotide oxidized form; NADH, Nicotinamide adenine dinucleotide reduced form; OXPHOS, oxidative phosphorylation.

Metformin and acetaminophen are widely used drugs which, similarly to statins, have been reported to inhibit the NADH-supported mitochondrial respiration and this unfavorable effect has been recently overcome by the use of cell-permeable succinate [19,20]. Similarly, the NADH-linked mitochondrial respiratory impairment caused by cerivastatin and atorvastatin in the present study was counteracted by means of a cell-permeable succinate prodrug, NV118 that bypassed the mitochondrial dysfunction and recovered the coupled (ATP-generating) respiration. The beneficial effects of the novel cell-permeable succinate compound are depicted in Figure 6.

Mitochondrial complex II oxidizes succinate and allows the transfer of electrons within the ETS which in turn allows the translocation of protons through complex III and IV leading to the establishment of a proton gradient across the mitochondrial inner-membrane and the subsequent proton transport through complex V with the generation of ATP [19]. Studies in isolated mitochondria in the LEAK state have shown that when supplied with saturating levels of succinate part of the electron flow is reversed and travels back through complex I generating reactive oxygen species (ROS) [56]. However, the risk of ROS increases through this mechanism seem to be low in the presence of complex I inhibition, which is exactly when cell-permeable succinates should be used [20,57].

Cell-permeable succinates can be used as a means to stimulate succinate supported respiration, eliminating the need for electron transfer through the impaired mitochondrial complex I whether it is induced by statins or other compounds, as has been shown previously [18,19,20]. As such, this makes cell-permeable succinates promising candidates for the treatment of SAMS.

## 4. Materials and Methods

### 4.1. Chemicals and Human Samples

All chemicals were obtained from Sigma-Aldrich (Saint Louis, MO, USA). The cell-permeable succinate prodrug was kindly provided by Abliva AB (Lund, Sweden) [18], and is also available via Oroboros Instruments in the MitoKit-CII (https://www.oroboros.at/index.php/product/mitokit-cii/).

Platelets were isolated from venous blood from healthy volunteers both men and women, aged between 27–32 (with one exception, a female aged 66), drawn in K_2_EDTA tubes according to a previously described protocol [15]. In brief, platelets were subjected to a sequence of differential centrifugations; the first at 500× *g* for 10 min and the second at 4600× *g* for 5–10 min.

### 4.2. Mitochondrial Respiration

Mitochondrial respiration was assessed by high-resolution respirometry using O2k-FluoRespirometers (Oroboros Instruments GmbH, Innsbruck, Austria) and a buffer (MiR05) containing: 0.5 mM EGTA, 3 mM MgCl_2_, 60 mM K-lactobionate, 20 mM taurine, 10 mM KH_2_PO_4_, 20 mM HEPES,110 mM sucrose and 1 g/L bovine serum albumin [58]. Experiments with human platelets were performed at 200 × 10^6^cells/mL at 37 °C.

The acute effects of statin exposure on mitochondrial respiration were evaluated by means of four different protocols:

Protocol I—Intact cells. Intact human platelets were subjected to Carbonyl cyanide-4-(trifluoromethoxy)phenylhydrazone(FCCP)-induced sub-maximal uncoupling (2 µM), to increase the resolution of the potential negative effects of statins on mitochondrial respiration, as compared to control (Dimethyl sulfoxide-DMSO). After the addition of FCCP, increasing concentrations of statins were titrated into the chamber (from 20 to 320 µM). In order to correct for the contribution of non-mitochondrial respiration, complex I inhibition was obtained with rotenone (2 µM) and complex III inhibition with antimycin A (1 µg/mL). The same experiments were repeated on a line of HepG2 cells using atorvastatin in order to assess replicability in other cells.

Protocol II—Permeabilized cells. Mitochondrial respiration in permeabilized human platelets was measured in the presence of increasing statin concentrations (40, 80, and 160 µM) and compared to DMSO (volume corresponding to the one used at the highest statin concentration), using a previously established protocol [15]. The concentrations were chosen after running a series of pilot experiments to identify the concentrations affecting mitochondrial function (data not shown).

Protocol III—Permeabilized mitochondria. Evaluation of the effect of statins on NADH-induced oxygen consumption. In order to ascertain whether the statins directly or indirectly inhibit complex I (NADH-dehydrogenase), platelet mitochondria were exposed to NADH before and after the addition of the highest concentration of statin that had been shown to decrease mitochondrial respiration in previous experiments. Specifically, permeabilized platelets with permeabilized mitochondria (digitonin, 1 µg/1 × 10^6^ platelets and alamethicin, 5 µg/mL, respectively) were exposed to the specific complex I substrate NADH (0.75 mM), followed by a single addition of statin (160 µM) vs. DMSO. Residual NADH-linked respiration was evaluated by a second addition of NADH with the same concentration in order to measure the difference in response of the mitochondrial respiration before and after statin exposure. Complex I was then inhibited using rotenone (2 µM).

Protocol IV—Intact cells and prodrug treatment. Intact platelet respiration was measured in the presence of a statin concentration of 80 µM (that elicited a clear decrease in mitochondrial respiration in the previous experiments). Cell-permeable succinate, NV118 (500 µM) or a DMSO control, was then added in an attempt to bypass mitochondrial complex I dysfunction. Coupled respiration was assessed using an ATP-synthase inhibitor, oligomycin (1 μg/mL), after which consecutive titrations of FCCP were added to achieve maximal activity of the ETS. To evaluate the degree of residual and/or unspecific, non-mitochondrial respiration (ROX) elicited by the prodrug or cellular oxidative side reactions remaining after the inhibition of the ETS, complex I was inhibited with rotenone (2 µM), complex III with antimycin A (1 µg/mL), and complex IV with sodium azide (10 mM). As control, mitochondrial respiration in platelets exposed to the volume of DMSO corresponding to the injection volume of the statin and of the prodrug, were measured.

The same experiments were repeated in a line of HepG2 cells using atorvastatin in order to assess replicability in other cells.

### 4.3. Data Analysis

Statistical analysis was performed using GraphPad PRISM software (GraphPad Software version 8.0; La Jolla, CA, USA). All data are expressed as mean ± SEM. To account for the presence of residual oxygen consumption all data were corrected for non-mitochondrial oxygen consumption [22]. Statistical analyses (one-way or two-way ANOVA with Bonferroni post hoc test) were performed on the antimycin-corrected data or rotenone-corrected data (*n* = 4–6 for the protocols using human platelets and *n* = 3 for the protocols using HepG2 cells).

## 5. Conclusions

Statins induce a concentration-dependent, two-pronged dysfunction of human mitochondria resulting in a severe impairment of the ATP-generating capability. The complex-I related inhibition of electron transfer can be compensated by intracellular delivery of the complex II substrate succinate. Since the current guidelines recommend the use of statins in the highest tolerated dose [2] and SAMS still represent the major reason for treatment discontinuation in medical practice [59], the use of a cell-permeable succinate could be a potential strategy in the acute treatment of severe statin-associated muscle symptoms and rhabdomyolysis.

## Figures and Tables

**Table 1:** Summary of the respiratory effects of the studied statins.

Effect	Simvastatin	Atorvastatin	Cerivastatin
OXPHOS coupling efficiency reduction	YES	YES	YES
ET capacity inhibition	YES	YES	YES
NADH-linked ETS inhibition	YES	YES	YES
Direct inhibition of NADH-dehydrogenase	YES	YES	NO
Succinate-linked ETS inhibition	YES	NO	NO
Uncoupling (increased LEAK respiration)	NO	YES	YES

## Data Availability

Data is contained within the article.

## Abbreviations

ADP	Adenosine diphosphate
ATP	Adenosine triphosphate
Cit. c	Cytochrome c
DMSO	Dimethyl sulfoxide
ET	Electron transport
ETS	Electron transport system
FCCP	Carbonyl cyanide p-(trifluoromethoxy) phenylhydrazone
LDL	Low-density lipoprotein
LEAK	Non-phosphorylating respiration
OXPHOS	Oxidative phosphorylation
NADH	Nicotinamide adenine dinucleotide reduced form
ROS	Reactive oxygen species
ROX	Residual oxygen consumption
SAMS	Statin-associated muscle symptoms
